# How the change of OMe substituent position affects the performance of spiro-OMeTAD in neutral and oxidized forms: theoretical approaches†

**DOI:** 10.1039/c8ra01879k

**Published:** 2018-05-18

**Authors:** Habib Ashassi-Sorkhabi, Parvin Salehi-Abar

**Affiliations:** Department of Physical Chemistry, Faculty of Chemistry, University of Tabriz Tabriz Iran habib.ashassi@gmail.com salehip_tabrizu@yahoo.com

## Abstract

Density functional theory (DFT) was used to investigate the electronic and optical properties of the *ortho*, *meta*, and *para* derivatives of 2,2′,7,7′-tetrakis-(*N*,*N*-di-4-methoxyphenylamino)-9,9′spirobifluorene (spiro-OMeTAD) and its two oxidized forms (+1 and +2). The energy level, distribution shape, and density of highest occupied molecular orbital (HOMO) and of lowest unoccupied molecular orbital (LUMO) were computed for all three derivatives and compared in the neutral and oxidized forms. Results indicated that the different positions of OMe in the spiro-OMeTAD framework lead to different optical properties. It was also found that compared to the neutral form, in the oxidized forms, the maximum absorption band red shifts, new signals in the visible range between 500 and 850 nm appear, and the Stokes shift values reduce for all three derivatives. The exciton binding energy of spiro-OMeTAD with an *o*-OMe substituent is 0.52 eV, being smaller than that of *p*-OMe and *m*-OMe, indicating easier generation of free charge carriers. The hole mobility was calculated for all three molecules, and the obtained data revealed that the hole mobility of the *o*-OMe substituent has a value of 7.90 × 10^−3^ cm^2^ V^−1^ s^−1^, which is respectively 3 and 11 times larger than that of *p*-OMe and *m*-OMe. The smaller exciton binding energy and larger hole mobility of the *o*-OMe substituent will result in a higher short-circuit current density (*J*_sc_) and a higher fill factor, respectively, demonstrating that *po*-spiro-OMeTAD is a promising candidate for use in perovskite solar cells. The reorganization energy, electron affinity, and ionization potential were also calculated and discussed.

## Abstract 2

Effect of changing the position of OMe substituent on the electrochemical and optical properties of spiro-MeOTAD hole conductor in neutral and oxidized forms for use in perovskite solar cells.

## Graphical abstract

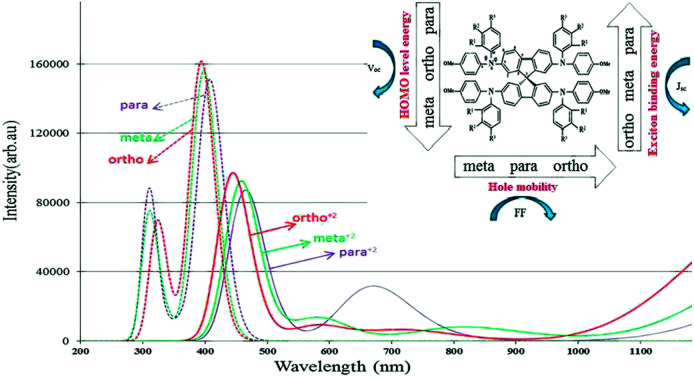

## 1. Introduction

In recent years, perovskite-absorber solar cells (PSCs) have become a critical aspect of research focusing on power conversion efficiency.^1^ Despite the considerable applicability of PSCs, decreasing device instability and large variations in the device performance caused by lack of reproducibility are the main limiting factors in this type of solar cell. Sanchez *et al.*^2^ demonstrated that the degradation of the hole transport materials (HTMs) is one of the reasons causing a loss in the cell performance. Consequently, the development of stable HTMs with minimal absorption in the visible and near-IR regions and with a good hole mobility is one of the critical issues to improve the efficiency of PSCs.^3^ To date, a great number of HTMs including inorganic, polymeric, and small organic molecules have been synthesized and applied in perovskite-based solar cells. Moreover, to design and develop an ideal p-type semiconducting material, some theoretical investigations have been carried out on the various organic structures as HTMs.^4–6^ However, among all the HTMs examined, 2,2′,7,7′-tetrakis-(*N*,*N*-di-4-methoxyphenylamino)-9,9′spirobifluorene (spiro-OMeTAD) exhibits good potential to be used as an efficient HTM.^7^ Nevertheless, the intrinsic hole mobility and conductivity of spiro-OMeTAD are low which require doping to be improved. On the other hand, the use of dopants leads to instability of device and the formation of oxidized forms, being clearly considered as a drawback for using spiro-OMeTAD as the HTM.^8^ The neutral spiro-MeOTAD absorbs light in the UV region, while its oxidized forms show relatively strong absorptions through the visible and near-IR ranges. Because absorption of the oxidized species occurs in the same spectral range where sensitizers absorb light, an unfavorable filtering effect is observed which could result in a reduced photocurrent. According to a brief literature survey, Nguyen *et al.*^9^ have experimentally investigated the optical properties and hole conductivity of dicationic salt of *para* position of spiro-OMeTAD, named as spiro(TFSI)2. In another experimental work reported by Jeon *et al.*,^10^ three spiro-OMeTAD derivatives have been synthesized and employed as a HTM to fabricate MAPbI_3_ perovskite solar cells. The results indicate that *o*-OMe derivative shows a high open-circuit voltage (1.02 V), a high fill factor (77.6%), a moderate short-circuit current density (21.2 mA cm^−2^), and an acceptable PCE (16.7%), while *p*-OMe and *m*-OMe derivatives show the PCEs of 14.9% and 13.9%, respectively. Fantacci *et al.*^11^ studied the opto-electrical properties of neutral *p*-OMe derivative of spiro-OMeTAD and its oxidized forms and found that their absorption spectra are respectively in the UV region and visible and near-IR ranges. Shi^12^ examined the crystal structure and hole transport mechanism of *p*-OMe derivative of spiro-OMeTAD. Murray *et al.*^13^ studied the electronic properties of spiro-OMeTAD analogues, and Chi *et al.*^5^ investigated the hole-transport properties of spiro-CPDT analogues. Some other theoretical studies were also performed on the hole-transport properties of spiro-MeOTAD and its related compounds.^14,15^ Although several experimental and theoretical papers have been published on *para* position of spiro-OMeTAD, there are no theoretical studies on the application of *ortho* and *meta* derivatives of spiro-OMeTAD as HTMs in perovskite-based solar cells. Hence, in the present work, due to the fact that changing the OMe substituent position in spiro-OMeTAD gives different band gaps and molecular orbital energy levels, we study the effect of different positions of the OMe substituent on the molecular orbitals, absorption and emission properties, and transport properties through density functional theory (DFT) and Time-Dependent DFT (TD-DFT) combined with the Marcus theory for both the neutral and oxidized states. We hope that the results reported herein should provide guidance in the design of new HTMs with higher power conversion efficiency and increased hole mobility.

## 2. Computational methods

Density functional theory was employed using Gaussian 03 software package to calculate the molecular structures of three spiro-OMeTAD derivatives in neutral and oxidized forms. For this purpose, we first used the B3LYP exchange-correlation functional and the 6-31G** basis set to optimize the neutral structures *in vacuo* and in chlorobenzene where the solvent effect was taken into account using the conductor-like polarizable continuum model (C-PCM) with considering the dielectric constant of chlorobenzene (*ε* = 5.6968). Frequency calculations were performed to ensure that the optimized structures are in minimum-energy points, where show no imaginary frequency. The oxidized forms were optimized by unrestricted (U) B3LYP/6-31G** level of theory. Time-dependent density functional theory (TD-DFT) was applied to determine the optimal geometries of electronically excited-states which are particularly useful to analyze the emission based on the B3LYP/6-31G** level.^11^

In this work, we theoretically investigate the hole transport behavior of the derivative with *o*-OMe substituent by the Marcus theory with hopping model. The charge hopping rate (*k*) is expressed as:^16^1
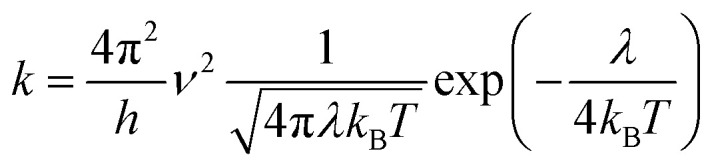
where *ν* is the transfer integral, *λ* is the reorganization energy, *h* is the Planck constant, *T* is the temperature in Kelvin, and *k*_B_ is the Boltzmann constant. The internal reorganization energy (*λ*) obtained from the adiabatic potential energy surface method can be expressed as follows:2
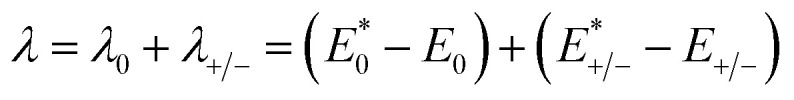
where 
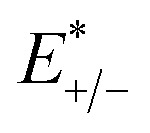
 and 
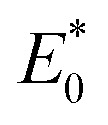
 represent the total energies of charged species and neutral species with the geometries of the neutral and charged species, respectively. *E*_+/−_ and *E*_0_ are the total energies of the charged species and neutral molecules in their lowest energy geometries, respectively. The transfer integral (*ν*) denotes the matrix element for the overlap of the initial and final state wave functions. Here, the transfer integral was investigated by adopting a direct approach at M06-2X/6-31G** level^17^ which can be written as:^18^3
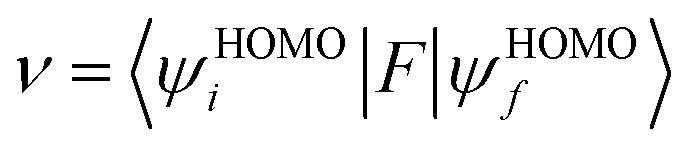
where *F* is the Kohn–Sham–Fock matrix for the dimer and *ψ*_*i*_ and *ψ*_*f*_ represent the frontier orbitals for hole transfer of isolated molecules 1 and 2 in the neutral dimer, respectively. The hole mobility of investigated molecules are calculated by using Einstein equation:^19,20^4
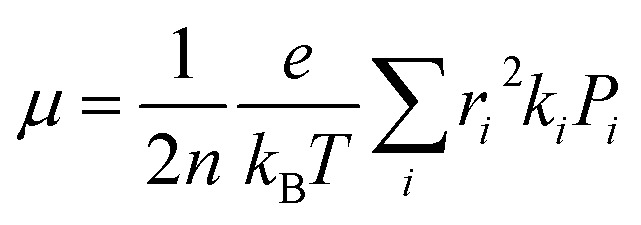
where *n* represents the spatial dimensionality and is 3 in our work, *i* is a selected hopping pathway, *r*_*i*_ is the charge hopping centroid to centroid distance, and *k*_*i*_ denotes the charge hopping rate. *P*_*i*_ is defined as the hopping probability, which can be obtained by the following equation:5
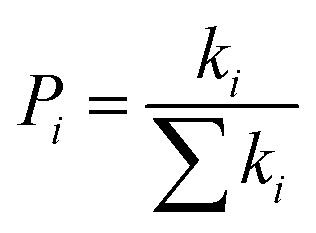


## 3. Results and discussion

### 3.1. Geometric and electronic structure

**Fig. 1:**
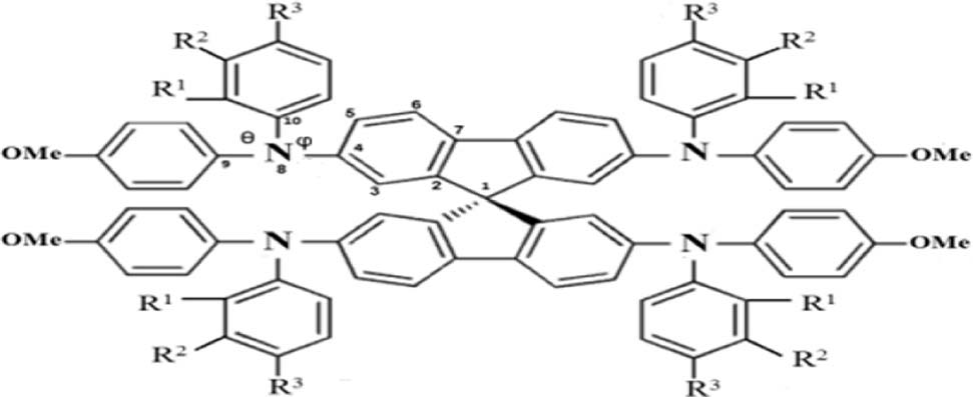
The chemical structure of spiro-OMeTAD derivatives. *o*: R_1_ = OMe, R_2_ = H, and R_3_ = H; *m*: R_1_ = H, R_2_ = OMe, and R_3_ = H; *p*: R_1_ = H and R_2_ = R_3_ = OMe.

**Fig. 2:**
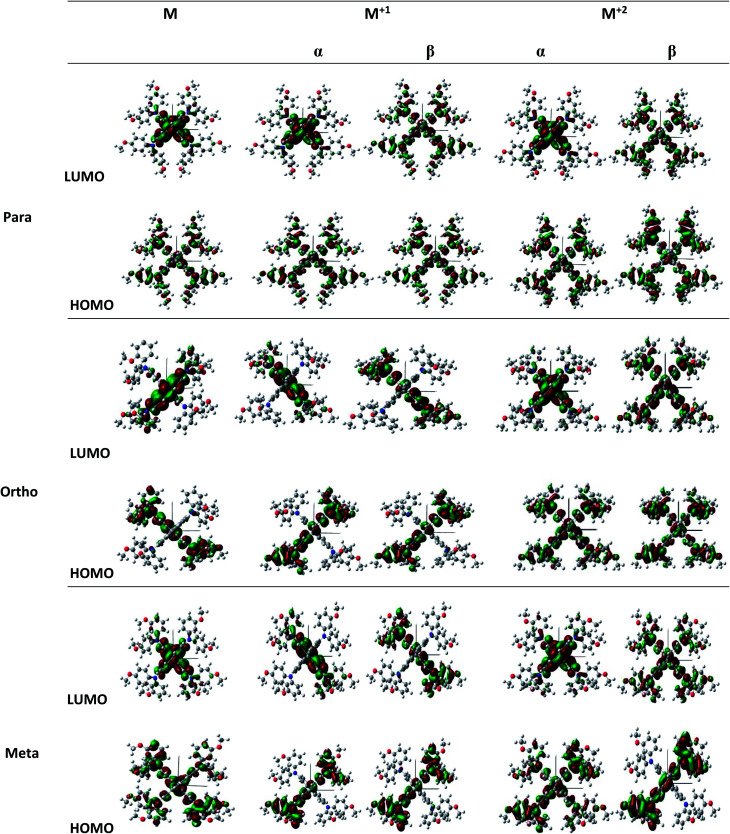
Illustration of frontier molecule orbitals of *pp*-, *pm*-, and *op*-spiro-OMeTAD at the B3LYP/6-31G** level for M and M^+1^/M^+2^ states.

Structures of the spiro-OMeTAD derivatives are shown in Fig. 1. The data regarding the optimized geometric structures of the spiro-OMeTAD in its neutral and oxidized forms are also given in Table 1. Obviously, removal of the electron from HOMO does not show any noticeable effect on the bond length distance of all the oxidized structures relative to the neutral state. The main difference between the neutral and oxidized forms is in the value of dihedral angle between the OMe-substituted phenyls and the fluorene ring (*Φ*) which increases upon oxidation. It can be found that the trend of *Φ* in the neutral form is as *p*- > *m*- > *o*-OMe-phenyl groups and in all the oxidized forms is as *o*- > *m*- > *p*-OMe-phenyl groups. To gain insight into the electronic structure, HOMOs and LUMOs of the three molecules investigated in neutral and oxidized forms are shown in Fig. 2. The distribution shape and the HOMO energy level in each derivative represent respectively the density of states involved in hole transport and the local energy of a positive charge for a site of particular geometry. The good HOMO delocalization and the proper HOMO energy level relative to the valence band of perovskite is favorable to enhance the hole transfer integral and the hole transport. In the neutral form, the HOMOs of *pp*- and *pm*-spiro-OMeTAD are delocalized approximately over the whole molecule and their LUMOs are predominantly localized on spiro-unit, while the HOMO of *po*-spiro-OMeTAD is localized on two (out of four) of the triphenylamine groups and its LUMO is mainly localized on the central part of the two triphenylamine groups. The FMO analysis reveals that the HOMO of the three derivatives in the neutral form is distributed more widely than their LUMO, showing that the neutral forms of the studied derivatives have a proper potential to be used as hole transfer materials.

**Table 1:** The optimized geometrical parameters of the neutral (M) and +1 (M^+^) and +2 (M^+2^) oxidized forms of spiro-OMeTAD (the bond distances are in angstrom and the angles are in degree)

Parameter	M			M^+^			M^+2^		
	*Para*	*Ortho*	*Meta*	*Para*	*Ortho*	*Meta*	*Para*	*Ortho*	*Meta*
C_1_–C_2_	1.539	1.535	1.535	1.541	1.532	1.532	1.535	1.534	1.535
C_2_–C_3_	1.386	1.386	1.386	1.382	1.375	1.376	1.376	1.375	1.376
C_3_–C_4_	1.410	1.408	1.408	1.414	1.421	1.417	1.418	1.422	1.419
C_4_–C_5_	1.410	1.409	1.408	1.415	1.425	1.421	1.420	1.424	1.421
C_5_–C_6_	1.393	1.394	1.393	1.388	1.380	1.382	1.383	1.380	1.382
C_6_–C_7_	1.398	1.399	1.398	1.401	1.408	1.407	1.406	1.408	1.409
C_7_–C_2_	1.408	1.408	1.408	1.412	1.421	1.419	1.417	1.420	1.418
C_4_–N_8_	1.416	1.419	1.421	1.405	1.387	1.395	1.395	1.386	1.394
N_8_–C_9_	1.426	1.424	1.428	1.427	1.428	1.429	1.428	1.427	1.428
N_8_–C_10_	1.422	1.422	1.416	1.424	1.432	1.425	1.423	1.433	1.426
*θ*(C_10_–N_8_–C_9_)	119.563	119.918	120.352	119.057	117.786	119.140	118.993	117.802	119.069
*Φ*(C_3_–C_4_–N_8_–C_10_)	142.302	133.209	136.829	146.057	154.107	147.347	147.805	154.231	148.666

The unrestricted calculation in the oxidized forms, which has a multiplicity other than one, produces two complete sets of orbitals including α and β manifolds that have different spatial wave functions and therefore different energy levels. The distribution shape of FMOs differs from alpha to beta orbitals. For *para* position, the α distribution shape and density of FMOs in the M^+1^ and M^+2^ oxidized forms are almost similar to those in the neutral form. However, the β distribution shape of FMO in the oxidized forms is quite different from that in the neutral form, and both of LUMO and HOMO in the oxidized forms are delocalized approximately over the whole molecule. For *ortho* position, the α distribution density of FMO in M^+1^ is similar to that in the neutral form, and the β distribution density of LUMO is higher than that of the neutral form. Additionally, in the M^+2^ oxidized form, the α and β distribution densities of FMO increase compared to the neutral form. For *meta* position, the α and β distribution densities of HOMO in M^+1^ are lower than those in the neutral form. Moreover, the α distribution density and shape of FMO in the M^+2^ oxidized form are similar to those in the neutral form, while the β distribution density of HOMO and LUMO in M^+2^ is respectively lower and higher than that in the neutral form.

It is obvious that a good delocalization of HOMO is favorable to enhance the hole transfer integral. Accordingly, it can be predicted from Fig. 2 that the oxidized structures with *ortho* and *para* positions will exhibit very weak performance due to the similarity of the distribution density of HOMO and LUMO. Furthermore, the oxidized structures with *meta* position cannot practically act as HTMs because of the fact that the distribution density of LUMO in these structures is higher than HOMO.

As it is clear from Table 2, although we cannot obtain the accurate HOMO and LUMO energy levels by DFT, the variation trend between the calculated and experimental values are in agreement. Hammett demonstrates that the OMe substituent at *meta* and *para* positions has electron-withdrawing and electron-donating effects, respectively.^21^ The HOMO energy levels of *po*- and *pm*-spiro-OMeTAD are deeper respect to *pp*-spiro-OMeTAD, which this will facilitate the injection of holes. The deeper HOMO levels of HTMs explain the high open-circuit voltage (*V*_oc_) of the PSC, because the *V*_oc_ of a solar cell is related to the difference between the quasi-Fermi levels of TiO_2_ and the HOMO energy level of HTM.^22,23^ Furthermore, the LUMO energy levels of *pm*- and *po*-spiro-OMeTAD are respectively lower and higher than those of *pp*-spiro-OMeTAD. According to these results, it can be concluded that due to the electron withdrawing effects, *m*-OMe substituent has lower molecular orbital energy level compared to *p*-OMe substituent. The band gap energies of *po*- and *pm*-spiro-OMeTAD are higher than those of *pp*-spiro-OMeTAD representing their high stability owing to the fact that in chemical reactions, the reactivity at *ortho* and *meta* positions of spiro-OMeTAD are lower than that at *para* position.^24^ The higher LUMO energy level of the HTM than the conduction band for perovskite can properly block the transfer of electron in perovskite back to *meta*l electrodes. Therefore, the *ortho*-substituted derivative may be a more suitable HTM than the *para* and *meta* due to its higher LUMO energy level relative to the two other derivatives.^25^

**Table 2:** The FMO energies and band gaps for the molecules M and M^+1^/M^+2^ at the B3LYP/6-31G** and UB3LYP/6-31G** levels, respectively (the experimental data in brackets has been taken from ref. 10)

Derivatives	M				M^+1^			M^+2^		
	HOMO (eV)	LUMO (eV)	Band gap		HOMO (eV)	LUMO (eV)	Band gap	HOMO (eV)	LUMO (eV)	Band gap
*Para*	−4.21(−5.22)	−0.66(−2.28)	3.55(2.94)	α	−6.34	−2.87	3.47	−8.45	−5.07	3.38
				β	−6.06	−5.67	0.39	−8.34	−7.52	0.82
*Ortho*	−4.24(−5.22)	−0.61(−2.18)	3.63(3.01)	α	−6.40	−2.86	3.54	−8.55	−4.98	3.57
				β	−6.15	−5.72	0.43	−8.59	−7.56	1.03
*Meta*	−4.36(−5.31)	−0.75(−2.31)	3.61(3.0)	α	−6.55	−3.00	3.55	−8.71	−5.23	3.48
				β	−6.27	−5.84	0.43	−8.68	−7.74	0.94

Removal of electron from β manifolds leads to the M^+^ and M^+2^ oxidized forms. Based on Table 2, the HOMO level of the *para*, *ortho*, and *meta* for M^+^ is −6.06, −6.15, and −6.27 eV, respectively, and these values for M^+2^ are −8.34, −8.59, and −8.68 eV. Accordingly, the HOMO levels of the oxidized forms are much lower than those of the neutral form and are lower than the perovskite valence band. This makes the effective hole injection impossible from perovskite to the oxidized forms of HTM, leading to a reduction in *V*_oc_ and consequently very low PCE in the oxidized forms.

### 3.2. Absorption spectrum

**Fig. 3:**
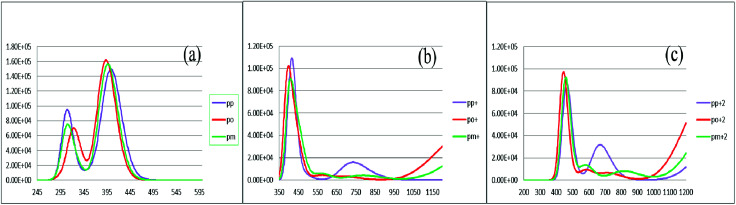
The calculated absorption spectra of the three derivatives of spiro-OMeTAD in the neutral (a), +1 (M^+^) (b), and +2 (M^+2^) oxidized forms (c) at the TDB3LYP/6-31G** level in chlorobenzene.

**Fig. 4:**
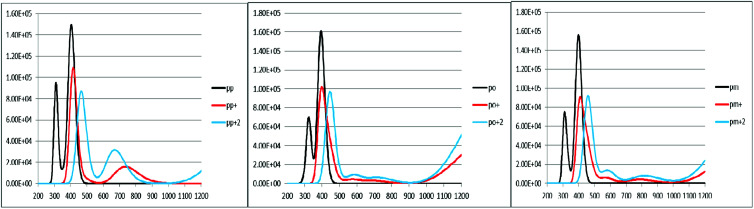
A comparison between the absorption spectra and energies of the electronic transitions of the spiro-OMeTAD derivatives in the neutral (M) and 1+ (M^+^)/2+ (M^2+^) oxidized forms.

To characterize the optical properties, the calculations were performed to simulate the absorption and emission spectra on the optimized ground and excited state geometries, respectively. The absorption spectra of the neutral and oxidized forms of the three derivatives of spiro-OMeTAD in chlorobenzene are shown in Fig. 3. Moreover, the wavelength of maximum absorbance (*λ*^max^_abs_), maximum emission (*λ*^max^_em_), and computed Stokes shifts are reported in Table 3. It is clear from this table that the calculated *λ*^max^_abs_ values of *pp*-, *po*-, and *pm*-spiro-OMeTAD in chlorobenzene are 401.60, 393.11, and 397.09 nm, respectively, which originate from π → π* transition and HOMO/HOMO −1 to both LUMO and LUMO +1 transitions. This trend in the absorption is similar to that found in the experimental reports.^10^ Compared to the *para* and *meta*, the *ortho* has larger oscillator strengths and its *λ*^max^_abs_ exhibits a slight blue shift. The emission spectra of all the three derivatives originate from π* → π and LUMO → HOMO transitions. The origin of these transitions containing the starting and arriving states has been defined using the analysis of the TD-DFT eigenvectors, which is reported in Tables S1–S9.† The *λ*^max^_em_ values of the *para*, *ortho*, and *meta* are 463.09, 449.72, and 449.62 nm, respectively, indicating that the *λ*^max^_em_ of the *para* is slightly red shifted by 13.37 and 13.47 nm as compared to *ortho* and *meta* positions. For a better comparison, the absorption spectra of the neutral and oxidized species of the three derivatives of spiro-OMeTAD are plotted in Fig. 4.

**Table 3:** The wavelength of maximum absorbance *λ*^max^_abs_ (nm) and maximum emission *λ*^max^_em_ (nm) of the spiro-OMeTAD derivatives in the neutral (M), 1+ (M^+^) and 2+ (M^+2^) oxidized forms in chlorobenzene based on the S_0_ and S_1_ states, respectively along with the Stokes shift at the TD-B3LYP/6-31G** level

Compound	*λ* ^max^_abs_ (nm)	*f*_abs_	*λ* ^max^_em_ (nm)	*f*_em_	Shift
**M**					
*Para*	401.60	1.1368	463.09	0.9254	61.49
*Ortho*	393.11	1.2078	449.72	1.0011	56.61
*Meta*	397.09	1.0715	449.62	1.0666	52.53

**M** ^**+**^					
*Para*	415.17	0.5377	426.41	0.8437	11.24
*Ortho*	392.70	1.1512	394.99	1.1393	2.29
*Meta*	403.88	0.8273	405.71	0.9533	1.83

**M** ^**+2**^					
*Para*	470.93	0.3188	435.75	0.4891	−35.18
*Ortho*	450.62	0.3468	417.33	0.4904	−33.29
*Meta*	458.02	0.6303	429.75	0.4633	−28.27

In the oxidized forms, the *λ*^max^_abs_ red shifts with respect to the neutral species with the sequence of relative intensity as M > M^+^ > M^+2^ for *para* and *ortho* positions and the order of M > M^+^ ∼ M^+2^ for *meta* position of spiro-OMeTAD. The new signals appear in the visible range between 500 and 850 nm with the sequence of intensity as M^+2^ > M^+^, reflecting the formation of the oxidized forms of the spiro derivatives, which might compete with the sensitizer in absorbing light leading to a decrease in the photovoltaic performance of PSCs. The data reported here on the oxidized states are important because most of the previously published experimental and computational works discuss the formation of the radical cation of *para* position,^11,26–28^ but there is no work reporting on the oxidized forms of *meta* and *ortho* positions of spiro-OMeTAD.

**Fig. 5:**
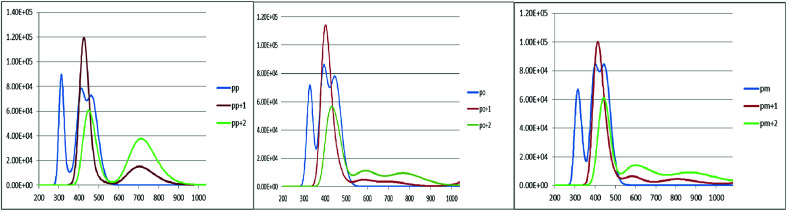
The calculated emission spectra of the three derivatives of spiro-OMeTAD in the neutral (M) and +1 (M^+^)/+2 (M^+2^) oxidized forms at the TDB3LYP/6-31G** level in chlorobenzene.

An increase in the difference between the ground and excited states geometry of the molecules makes the Stokes shifts increase, causing a flexibility in the structures and an enhancement in the pore-filling of HTMs.^29^ The *para* in the neutral form has the Stokes shift of 61.49 nm, which is larger than that of the *ortho* and *meta* positions with the values of 56.61 and 52.53 nm, respectively. Most interestingly, the Stokes shifts of the *para*, *ortho*, and *meta* in the +1 oxidized form are respectively 11.24, 2.29, and 1.83 smaller than those in the neutral form, while their Stokes shifts in the +2 oxidized form have negative values (see Table 3). Additionally, according to Fig. 5, intensity of the emission spectra of the three derivatives in the 1+ and +2 oxidized forms are respectively higher and lower than those in the neutral form. From these observations, it seems that the recombination process increases in the +1 oxidized form due to the increased intensity of the emission spectra.

### 3.3. Reorganization energy, electron affinity, and ionization potential

Reorganization energy is an important factor to investigate the charge transport behavior of HTMs. This character is related to the change occurring in the dihedral angle during the charge transfer. The variation tendencies of reorganization energy and hole transfer integral are opposite. The reorganization energies of holes (*λ*_h_) are given in Table 4. The calculated *λ*_h_ values of the *para*, *ortho*, and *meta* in chlorobenzene are 0.11, 0.22, and 0.20 eV, respectively. Clearly, the *λ*_h_ values decrease with the increase of electron-donating ability of substituent groups. It has already been mentioned that the *p*- and *o*-OMe substituents have an electron-donating effect and the *meta* expresses an electron-withdrawing effect. Furthermore, the *λ*_h_ of the *ortho* is higher than that of the *m*-OMe substituent by 0.02 eV, which is mainly caused by the difference of dihedral angle between the -OMe substituted phenyls and the fluorene ring (*Φ*) in the neutral and cation forms. The difference of the *Φ* dihedral angle between the neutral and cation states of the *pp*-, *po*-, and *pm*-spiro-OMeTAD is 3.75°, 20.89°, and 10.518°, respectively. The *ortho* position has the largest structure distortion between the neutral state and the cation state and needs more reorganization energy. Adiabatic ionization potential is a useful parameter to evaluate the stability of hole transport materials in the term of resistance to oxidization. To the best our knowledge, having high adiabatic ionization potentials (IP_a_) usually results in relatively more stability.^15^ The adiabatic ionization potentials and adiabatic electron affinities (EA_a_) of the three derivatives studied were calculated by IP_a_ = (*E*_+_ − *E*_0_) and EA_a_ = (*E*_0_ − *E*_*−*_), and the results are summarized in Table 4. Obviously, the IP_a_ and EA_a_ of the *para* position have the lowest values due to the increased energy of the resonance system. In the case of the *meta* position, contribution of the oxygen donation is lower, which leads to an increase in both IP_a_ and the EA_a_.^30^ Moreover, substitution at the *ortho* position compared to the *para* does not show a significant change in the IP_a_, meaning that the electron donating property of the OMe group is maintained in the *ortho* position. Accordingly, it could be concluded that the *o*- and *p*-OMe have almost the same electron donating effects.

**Table 4:** The internal hole reorganization energies (*λ*_h_, eV), the adiabatic ionization potentials (IP_a_, eV), and the adiabatic electron affinities (EA_a_, eV) calculated at the B3LYP/6-31G** level

Derivative	*λ*_h_	IP_a_	EA_a_	*η*
*Para*	0.11	4.47	1.07	1.71
*Ortho*	0.22	4.46	1.08	1.70
*Meta*	0.20	4.59	1.12	1.74

Stability of the structures investigated is another important factor that has to be considered and indicated by absolute hardness (*η*) as *η* = (IP_a_ − EA_a_)/2. The *η* values of the structures were calculated and listed in Table 4. From this table, we can conclude that among the three structures studied, the spiro-OMeTAD with the *meta* position has the largest stability. This most stable structure with the lowest energy levels will hinder changing of the number of electrons and increase the stability of materials, which is much more desirable for the molecular and materials design.^31,32^

### 3.4. Exciton binding energies and charge-transfer integrals

Strength of the electron–hole interaction is characterized by the exciton binding energy, which is one of the key parameters controlling the charge separation to escape from the coulombic attraction and is directly related to the estimation of the short-circuit current density (*J*_sc_) of solar cells. The exciton binding energy (*E*_b_) is defined as the potential energy difference between the neutral singlet exciton and the two free charge carriers and can be expressed as *E*_b_ = *E*_g_ − *E*_x_ = Δ_H–L_ − *E*_1_. In this equation, *E*_g_ is the electronic band gap and can be replaced by the energy gap (Δ_H–L_), and *E*_x_ is the optical gap and is generally defined as the first singlet excitation energy (*E*_1_).^33,34^ An easy charge separation from coulombic attraction leads to a low *E*_b_, resulting in easier electron–hole dissociation. The values of *E*_1_ and *E*_b_ for the examined systems are reported in Table 5. According to this table, the calculated exciton binding energies of *pp*-, *po*-, and *pm*-spiro-OMeTAD are 0.51, 0.49, and 0.50 eV, respectively. Inspection of these values indicates that the *po*-spiro-OMeTAD has the lowest *E*_b_, which allows easier electron–hole dissociation and subsequently results in a higher intramolecular charge transfer, enhancing the *J*_sc_ of solar cells.^15,35–37^ The variation trend in *J*_sc_ values predicted from the calculated results is consistent with the experimental reports where the *J*_sc_ of *ortho* and *meta* positions is larger than that of *para* position.

**Table 5:** The first singlet excitation energy (*E*_1_) and electron–hole binding energy (*E*_b_) calculated at the B3LYP/6-31G** level in chlorobenzene

Derivative	*E*_1_	*E*_b_
*Para*	3.04	0.51
*Ortho*	3.14	0.49
*Meta*	3.11	0.50

**Fig. 6:**
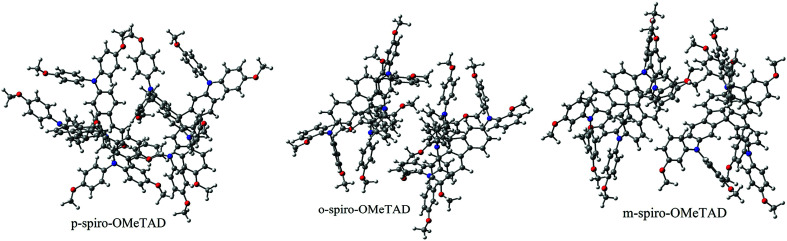
The optimized dimeric structures of the spiro-OMeTAD derivatives at B3LYP/6-31G** level.

The transfer integral (*v*) is a critical parameter to evaluate the charger-carrier mobility of HTMs. It is well known that to achieve a high hole mobility, the *v* needs to be maximized.^16^ Transfer integral depends on the overlapping degree and the center-of-mass distance of the interacting systems. In this work, our strategy to generate the transfer integrals of the structures studied is that two molecules of the obtained ground state from each structure were placed against each other in different directions and in centroid to centroid distance to get a reasonable guess for a stable dimer.^14^ The most stable dimer of all the three spiro-OMeTAD derivatives was obtained by using Gaussian 03 program package at the B3LYP/6-31G** level. It should be noted that after relaxation to their minimum potential energy, the centroid to centroid distance of dimer of *pp*-, *po*-, and *pm*-spiro-OMeTAD was different and obtained as 10.05, 9.43, and 10.71 Å, respectively. The stable dimers to calculate the hole transfer integral are shown in Fig. 6. The transfer integral between two molecules in the most stable dimer is obtained by adopting a direct approach at M06-2X/6-31G** level. The M06-2X functional, compared with 12 other functionals and the Hartree−Fock theory, is suitable for noncovalent interactions.^17^ The hole-hopping rate is also determined by eqn (1). The centroid-to-centroid distances of the stable dimer, hole transfer integrals, and hole-hopping rates are listed in Table 6. The results show that the *ortho* position functions as a more effective HTM than the two other positions due to its higher hole mobility resulting from a large hole transfer integral and a large hole hopping rate.

**Table 6:** The centroid to centroid distances (*r*_*i*_, Å), hole transfer integrals *v* (eV), hole hopping rates (*k*, s^−1^), and hole mobilities (*μ*, cm^2^ v^−1^ s^−1^) of the three spiro-OMeTAD derivatives

Derivative	*r*	*v*	*k*	*μ*
*Para*	10.05	1.47 × 10^−3^	3.77 × 10^10^	2.47 × 10^−3^
*Ortho*	9.43	5.70 × 10^−3^	1.37 × 10^11^	7.90 × 10^−3^
*Meta*	10.71	1.34 × 10^−3^	9.62 × 10^9^	7.16 × 10^−4^

Some studies have demonstrated that the high fill factor of PSC originates from the fast hole mobility.^38,39^ In the previous section, it was mentioned that reorganization energy of the *ortho* position is higher than that of the *para* and *meta* positions, resulting in a negative contribution to the hole mobility of the *ortho*. However, the two properties of transfer integral and reorganization energy offset each other. Accordingly, we can find a much faster hole mobility for the *ortho* owing to the dominating role of the transfer integral in the exponential term of eqn (1). Based on the experimental fill factor data reported for PSCs with the *pp*-, *po*-, and *pm*-spiro-OMeTAD as 71.1%, 77.6%, and 65.2%, respectively,^10^ it could be inferred that the hole mobility plays a key role in the determination of these parameters. It is worth noting that although the accurate hole mobilities cannot be obtained by this method, the relative order observed in the values of hole mobility to predict the fill factor is consistent with the order found for the experimental fill factors.

## 4. Conclusions

The results obtained from the FMO analysis show that in the neutral form, both of the *para* and *meta* derivatives have a similar distribution of the frontier orbitals, and HOMO is delocalized approximately over the whole molecule and LUMO is located on the spiro-unit. On the other hand, HOMO of the *ortho* derivate is localized on the two (out of four) of triphenylamine groups and its LUMO is mainly localized on the central part of the two triphenylamine groups. It was found that the oxidation of structures leads to a change in the distribution shape and reduces the density of HOMO to less than or equal to LUMO. Moreover, the decreased energy level of HOMO in the oxidized structures makes them inapplicable for use as efficient HTMs. The spectrum analysis indicates that in the oxidized forms, the maximum absorption band red shifts with respect to the neutral species, and new signals appear in the visible range between 500 and 850 nm. This phenomenon might compete with the sensitizer in absorbing light, resulting in a reduced photovoltaic performance of PSCs. Furthermore, the Stokes shifts of all the three derivatives in the +1 and +2 oxidized forms are smaller than those in the neutral form. Comparing the intensity of the emission spectrum in the neutral and oxidized forms, the increased intensity of the spectrum in the +1 form confirms the recombination process occurring in this oxidized form. Most importantly, in the neutral form, spiro-OMeTAD with *o*-OMe substituent has the lowest exciton binding energy with the value 0.52 eV and highest hole mobility with the value 7.90 × 10^−3^ cm^2^ V^−1^ s^−1^, demonstrating that the *o*-OMe substituent with higher short-circuit current density (*J*_sc_) and fill factor is a promising candidate for perovskite solar cells. We believe that the results reported herein should provide guidance in the design of new HTMs with higher power conversion efficiency and increased hole mobility.

## Conflicts of interest

There are no conflicts of interest to declare.

## Supplementary Material

RA-008-C8RA01879K-s001
