# Supplementary material for: How the change of OMe substituent position affects the performance of spiro-OMeTAD in neutral and oxidized forms: theoretical approaches
Source: RSC Adv. 2018 May 18;8(33):18234–42. doi: 10.1039/c8ra01879k (PMC9080512; doi:10.1039/c8ra01879k)
Supplement: RA-008-C8RA01879K-s001 [file RA-008-C8RA01879K-s001.pdf]

Electronic Supplementary Material (ESI) for RSC Advances.
This journal is © The Royal Society of Chemistry 2018

Supporting Information

How the change of OMe substituent position affects the performance of spiro-OMeTAD in neutral and oxidized forms: Theoretical approaches

Habib Ashassi-Sorkhabi* **and Parvin Salehi-Abar**

Department of Physical Chemistry, Faculty of Chemistry, University of Tabriz, Tabriz, Iran

∗ Corresponding author

Email address: habib.ashassi@gmail.com

salehip_tabrizu@yahoo.com

Table S1. Energy, wavelength, oscillator strength, and composition of the TDDFT excitations of "**pp**" in chlorobenzene.

States	Energy (eV)	Wavelength (nm)	F	Composition
1	3.0347	408.56	0.8957	(H-1->L+1 (25%), HOMO->LUMO (73%)
2	3.0873	401.60	1.1368	H-1->LUMO (52%), HOMO->L+1 (46%)
4	3.1486	393.78	0.0605	H-1->L+1 (74%), HOMO->LUMO (25%)
11	3.5255	351.68	0.0775	H-2->L+2 (11%), H-1->L+5 (16%), HOMO->L+4 (58%) H-3->L+3 (9%), H-2->L+1 (2%)
23	3.9669	312.55	0.1722	H-1->L+9 (34%), HOMO->L+8 (43%) ,H-3->L+3 (3%), H-3->L+9 (5%), H-2->L+8 (6%), HOMO->L+12 (2%)
24	3.9692	312.36	0.3021	H-1->L+8 (38%), HOMO->L+9 (38%) H-3->L+8 (6%), H-2->L+3 (2%), H-2->L+9 (6%), H-1->L+6 (3%), HOMO->L+7 (2%)
25	4.0064	309.46	0.0470	H-2->L+4 (10%), H-1->L+7 (37%), HOMO->L+6 (28%) H-1->L+11 (2%), HOMO->L+10 (7%), HOMO->L+12 (4%), HOMO->L+14 (3%)
27	4.0164	308.69	0.5159	H-2->L+4 (13%), H-1->L+13 (22%), HOMO->L+12 (38%), H-3->L+5 (2%), H-3->L+13 (3%), H-2->L+12 (3%), HOMO->L+6 (4%), HOMO->L+8 (4%)
30	4.0288	307.74	0.0996	H-3->L+4 (14%), H-1->L+12 (32%), HOMO->L+13 (30%), H-3->L+12 (3%), H-1->L+6 (3%), H-1->L+10 (2%)

Table S2. Energy, wavelength, oscillator strength, and composition of the TDDFT excitations of "**po**" in chlorobenzene.

States	Energy (eV)	Wavelength (nm)	F	Composition
1	3.1347	395.52	0.8752	H-1->LUMO (43%), HOMO->LUMO (44%), H-1->L+1 (3%), HOMO->L+1 (8%)
2	3.1539	393.11	1.2078	H-1->LUMO (28%), HOMO->L+1 (66%)
3	3.1963	387.90	0.1355	H-1->LUMO (24%), HOMO->LUMO (52%), HOMO->L+1 (22%)
10	3.6783	337.07	0.0456	H-3->LUMO (25%), H-2->LUMO (11%), H-2->L+1 (14%), HOMO->L+5 (29%), H-2->L+3 (5%), HOMO->L+4 (9%)
11	3.6972	335.34	0.0709	H-2->LUMO (11%), H-1->L+4 (33%), HOMO->L+5 (11%) H-3->LUMO (4%), H-3->L+2 (6%), H-2->L+1 (9%), H-2->L+3 (4%), HOMO->L+4 (4%), HOMO->L+6 (7%), HOMO->L+7 (4%)
12	3.6991	335.17	0.0476	H-2->LUMO (10%), H-1->L+4 (27%), HOMO->L+4 (23%) H-3->LUMO (3%), H-3->L+2 (9%), H-2->L+1 (5%), H-1->L+5 (2%), H-1->L+7 (5%), HOMO->L+6 (3%)
14	3.7569	330.02	0.0564	H-2->L+1 (22%), HOMO->L+4 (10%), HOMO->L+5 (31%), HOMO->L+6 (17%), H-2->L+5 (4%), HOMO->L+7 (4%), HOMO->L+9 (2%)
17	3.8305	323.68	0.1872	HOMO->L+6 (22%), HOMO->L+8 (14%), HOMO->L+9 (28%), H-2->L+6 (3%), H-1->L+8 (7%), HOMO->L+4 (6%), HOMO->L+5 (3%), HOMO->L+7 (6%)
18	3.8583	321.34	0.5286	H-1->L+7 (19%), H-1->L+8 (44%), HOMO->L+8 (10%) H-3->L+2 (2%), H-3->L+6 (3%), H-3->L+7 (3%), H-1->L+5 (2%), H-1->L+9 (7%), HOMO->L+7 (2%)

Table S3. Energy, wavelength, oscillator strength, and composition of the TDDFT excitations of "**pm**" in chlorobenzene.

States	Energy (eV)	Wavelength (nm)	F	Composition
1	3.1107	398.58	1.0370	H-1->LUMO (10%), H-1->L+1 (10%), HOMO->LUMO (63%), HOMO->L+1 (14%)
2	3.1224	397.09	1.0715	H-1->LUMO (24%), H-1->L+1 (26%), HOMO->L+1 (40%), HOMO->LUMO (9%)
13	3.7055	334.59	0.0790	H-2->L+3 (11%), H-1->L+4 (15%), HOMO->L+4 (34%), HOMO->L+5 (17%), H-2->L+2 (9%), H-1->L+5 (7%)
14	3.7129	333.93	0.0808	H-3->L+2 (11%), H-1->L+4 (31%), H-1->L+5 (15%), HOMO->L+4 (19%), HOMO->L+5 (10%) H-3->LUMO (2%), H-3->L+3 (8%)
19	3.9726	312.10	0.3101	H-2->L+3 (10%), H-1->L+8 (11%), HOMO->L+8 (28%) H-2->L+2 (9%), H-2->L+6 (9%), H-1->L+4 (3%), H-1->L+10 (2%), HOMO->L+4 (4%), HOMO->L+6 (4%), HOMO->L+7 (2%), HOMO->L+10 (5%)
20	3.9889	310.82	0.1376	H-1->L+11 (16%), HOMO->L+9 (20%), HOMO->L+10 (13%), H-3->L+2 (4%), H-3->L+3 (3%), H-3->L+7 (3%), H-2->L+3 (5%), H-2->L+8 (2%), H-1->L+5 (5%), H-1->L+6 (5%), H-1->L+9 (6%), HOMO->L+6 (3%), HOMO->L+8 (2%)
21	3.9976	310.15	0.1004	H-1->L+9 (20%), HOMO->L+6 (11%), HOMO->L+11 (11%) H-3->L+2 (9%), H-3->L+3 (7%), H-3->L+7 (3%), H-2->L+3 (2%), H-2->L+8 (4%), H-1->L+4 (3%), H-1->L+10 (3%), HOMO->L+5 (2%), HOMO->L+7 (2%), HOMO->L+8 (6%), HOMO->L+10 (6%)
22	4.0184	308.54	0.1182	H-2->L+2 (19%), H-2->L+3 (15%), HOMO->L+10 (19%), H-3->L+2 (5%), H-1->L+5 (3%), H-1->L+8 (8%), H-1->L+11 (8%), HOMO->L+4 (3%), HOMO->L+5 (2%), HOMO->L+8 (4%)
24	4.0371	307.11	0.2475	H-3->L+2 (11%), H-3->L+3 (11%), H-1->L+9 (19%), H-1->L+10 (14%), HOMO->L+9 (18%), H-3->L+10 (4%), H-1->L+5 (4%), HOMO->L+8 (4%), HOMO->L+10 (3%), HOMO->L+11 (2%)

Table S4. Energy, wavelength, oscillator strength, and composition of the TDDFT excitations of "**pp**$^{+}$" in chlorobenzene.

States	Energy (eV)	Wavelength (nm)	F	Composition
5	1.6277	761.71	0.0373	H-4(B)->LUMO(B) (98%)
6	1.6306	760.36	0.0481	H-5(B)->LUMO(B) (93%) H-8(B)->LUMO(B) (7%)
8	1.7070	726.33	0.0423	H-6(B)->LUMO(B) (98%)
9	1.7242	719.06	0.0914	H-8(B)->LUMO(B) (60%), H-7(B)->LUMO(B) (34%) H-5(B)->LUMO(B) (5%)
21	2.5491	486.39	0.0225	H-1(A)->LUMO(A) (15%), HOMO(A)->L+1(A) (15%), H-20(B)->LUMO(B) (22%), HOMO(B)->L+1(B) (35%)
22	2.5572	484.85	0.0288	H-1(A)->L+1(A) (11%), HOMO(A)->LUMO(A) (13%), H-21(B)->LUMO(B) (41%), HOMO(B)->L+2(B) (21%) H-19(B)->LUMO(B) (4%)
26	2.9482	420.55	0.0533	H-23(B)->LUMO(B) (63%) H-3(A)->L+1(A) (4%), H-2(A)->LUMO(A) (4%), HOMO(A)->LUMO(A) (2%), H-2(B)->L+1(B) (9%), H-1(B)->L+2(B) (7%), HOMO(B)->L+2(B) (6%)
27	2.9670	417.88	0.4904	H-1(A)->LUMO(A) (15%), HOMO(A)->L+1(A) (14%), H-2(B)->L+2(B) (11%), H-1(B)->L+1(B) (11%), HOMO(B)->L+1(B) (35%) H-3(A)->LUMO(A) (3%), H-2(A)->L+1(A) (3%), H-24(B)->LUMO(B) (3%)
28	2.9863	415.17	0.5377	H-1(A)->L+1(A) (13%), HOMO(A)->LUMO(A) (24%), H-23(B)->LUMO(B) (17%), HOMO(B)->L+2(B) (42%)
29	2.9991	413.40	0.3333	HOMO(A)->L+1(A) (11%), H-2(B)->L+2(B) (16%), H-1(B)->L+1(B) (22%), HOMO(B)->L+1(B) (12%) H-3(A)->LUMO(A) (7%), H-2(A)->L+1(A) (6%), H-1(A)->LUMO(A) (9%), H-24(B)->LUMO(B) (7%)
30	3.0160	411.09	0.0845	H-23(B)->LUMO(B) (18%), H-2(B)->L+1(B) (26%), H-1(B)->L+2(B) (22%) H-3(A)->L+1(A) (6%), H-2(A)->LUMO(A) (7%), H-1(A)->L+1(A) (2%), HOMO(A)->LUMO(A) (5%), HOMO(B)->L+2(B) (3%)

Table S5. Energy, wavelength, oscillator strength, and composition of the TDDFT excitations of "**po**$^+$" in chlorobenzene.

States	Energy (eV)	Wavelength (nm)	F	Composition
3	0.8702	1424.82	0.7067	H-2(B)->LUMO(B) (99%)
12	2.1579	574.57	0.0249	H-12(B)->LUMO(B) (30%), H-11(B)->LUMO(B) (56%) H-15(B)->LUMO(B) (4%), H-10(B)->LUMO(B) (2%), H-9(B)->LUMO(B) (5%)
13	2.1734	570.46	0.0285	H-12(B)->LUMO(B) (66%), H-11(B)->LUMO(B) (24%) H-15(B)->LUMO(B) (3%), H-14(B)->LUMO(B) (2%), H-9(B)->LUMO(B) (2%)
22	2.6907	460.78	0.0274	21702.10992 460.784690376 0.0274 2.547-A HOMO(A)->LUMO(A) (93%), H-2(A)->LUMO(A) (4%)
23	2.7437	451.88	0.2049	H-2(A)->LUMO(A) (36%), H-22(B)->LUMO(B) (17%), HOMO(B)->L+1(B) (25%), HOMO(A)->LUMO(A) (6%), H-23(B)->LUMO(B) (2%), H-21(B)->LUMO(B) (4%), H-19(B)->LUMO(B) (2%)
24	2.7704	447.53	0.0282	H-23(B)->LUMO(B) (25%), H-19(B)->LUMO(B) (26%), HOMO(B)->L+1(B) (10%), H-2(A)->LUMO(A) (6%), H-22(B)->LUMO(B) (7%), H-21(B)->LUMO(B) (9%), H-20(B)->LUMO(B) (7%), H-18(B)->LUMO(B) (2%)
26	2.8516	434.79	0.0289	H-23(B)->LUMO(B) (36%), H-22(B)->LUMO(B) (36%), HOMO(B)->L+1(B) (16%), H-21(B)->LUMO(B) (2%), H-19(B)->LUMO(B) (4%)
27	2.9283	423.41	0.4466	H-2(A)->LUMO(A) (38%), H-22(B)->LUMO(B) (12%), HOMO(B)->L+1(B) (41%), H-23(B)->LUMO(B) (2%)
31	3.1573	392.70	1.1512	HOMO(A)->L+2(A) (47%), HOMO(B)->L+2(B) (50%)
32	3.1682	391.34	0.0366	HOMO(B)->L+3(B) (67%), H-2(A)->L+1(A) (4%), H-1(A)->L+2(A) (2%), H-1(A)->L+9(A) (2%), HOMO(A)->L+2(A) (2%), HOMO(A)->L+9(A) (3%), H-1(B)->L+2(B) (2%), HOMO(B)->L+9(B) (2%), HOMO(B)->L+10(B) (2%)

Table S6. Energy, wavelength, oscillator strength, and composition of the TDDFT excitations of "**pm**$^{+}$" in chlorobenzene.

States	Energy (eV)	Wavelength (nm)	F	Composition
3	0.7702	1609.82	0.6796	H-2(B)->LUMO(B) (100%)
8	1.5895	780.02	0.0328	H-7(B)->LUMO(B) (19%), H-6(B)->LUMO(B) (72%) H-5(B)->LUMO(B) (6%)
13	2.1648	572.73	0.0682	H-12(B)->LUMO(B) (86%), H-13(B)->LUMO(B) (9%)
22	2.6223	472.81	0.0283	HOMO(A)->LUMO(A) (91%), H-2(A)->LUMO(A) (5%), HOMO(B)->L+1(B) (2%)
23	2.6712	464.16	0.1990	H-2(A)->LUMO(A) (42%), HOMO(B)->L+1(B) (40%) HOMO(A)->LUMO(A) (7%), H-21(B)->LUMO(B) (2%)
25	2.7936	443.81	0.0216	H-19(B)->LUMO(B) (83%), H-21(B)->LUMO(B) (6%), H-20(B)->LUMO(B) (4%), HOMO(B)->L+1(B) (3%)
26	2.8050	442.02	0.3143	H-2(A)->LUMO(A) (25%), H-22(B)->LUMO(B) (23%), HOMO(B)->L+1(B) (41%),H-19(B)->LUMO(B) (3%)
29	3.0643	404.61	0.2476	HOMO(A)->L+4(A) (25%), HOMO(B)->L+2(B) (15%), HOMO(B)->L+4(B) (28%), H-1(A)->L+1(A) (3%), HOMO(A)->L+1(A) (8%), HOMO(A)->L+3(A) (4%), H-1(B)->L+2(B) (3%)
30	3.0698	403.88	0.8273	HOMO(A)->L+1(A) (39%), HOMO(B)->L+2(B) (34%), HOMO(B)->L+4(B) (10%),HOMO(A)->L+4(A) (7%)

Table S7. Energy, wavelength, oscillator strength, and composition of the TDDFT excitations of "**pp**$^{+2}$" in chlorobenzene.

States	Energy (eV)	Wavelength (nm)	F	Composition
3	0.6913	1793.56	0.6417	H-1(B)->L+1(B) (58%), HOMO(B)->LUMO(B) (42%)
4	0.7128	1739.31	0.7800	H-1(B)->LUMO(B) (47%), HOMO(B)->L+1(B) (54%)
7	1.8221	680.43	0.0310	H-4(B)->L+1(B) (17%), H-3(B)->LUMO(B) (78%)
8	1.8234	679.95	0.1087	H-4(B)->LUMO(B) (25%), H-3(B)->L+1(B) (61%)
9	1.8370	674.94	0.0346	H-6(B)->LUMO(B) (54%), H-5(B)->L+1(B) (38%) H-4(B)->LUMO(B) (3%), H-3(B)->L+1(B) (5%)
10	1.8433	672.61	0.0200	H-7(B)->LUMO(B) (10%), H-6(B)->L+1(B) (30%), H-5(B)->LUMO(B) (54%) H-4(B)->L+1(B) (3%)
11	1.8579	667.34	0.0528	H-7(B)->LUMO(B) (62%), H-4(B)->L+1(B) (20%) H-6(B)->L+1(B) (8%), H-5(B)->LUMO(B) (7%)
12	1.8671	664.04	0.1893	H-7(B)->L+1(B) (53%), H-4(B)->LUMO(B) (36%) H-3(B)->L+1(B) (5%), H-2(B)->LUMO(B) (3%)
27	2.6303	471.37	0.2889	H-1(A)->L+1(A) (16%), HOMO(A)->LUMO(A) (20%), H-17(B)->LUMO(B) (24%), H-16(B)->L+1(B) (25%) H-15(B)->LUMO(B) (8%)
28	2.6328	470.93	0.3188	H-1(A)->LUMO(A) (16%), HOMO(A)->L+1(A) (17%), H-17(B)->L+1(B) (20%), H-16(B)->LUMO(B) (33%) H-15(B)->L+1(B) (6%)
29	2.6636	465.47	0.0227	H-18(B)->L+1(B) (28%), H-15(B)->LUMO(B) (52%) H-1(A)->L+1(A) (2%), HOMO(A)->LUMO(A) (3%), H-17(B)->LUMO(B) (5%), H-14(B)->L+1(B) (2%), H-13(B)->LUMO(B) (6%)
30	2.6699	464.38	0.0327	H-18(B)->LUMO(B) (29%), H-15(B)->L+1(B) (27%), H-14(B)->LUMO(B) (13%), H-13(B)->L+1(B) (13%) H-1(A)->LUMO(A) (2%), HOMO(A)->L+1(A) (2%), H-19(B)->L+1(B) (6%), H-17(B)->L+1(B) (5%)
31	2.6773	463.10	0.0228	H-18(B)->LUMO(B) (13%), H-15(B)->L+1(B) (18%), H-14(B)->LUMO(B) (36%), H-13(B)->L+1(B) (11%) H-19(B)->L+1(B) (9%), H-12(B)->LUMO(B) (2%), H-11(B)->L+1(B) (2%), H-9(B)->L+1(B) (3%)
33	2.6976	459.62	0.1013	H-20(B)->L+1(B) (26%), H-19(B)->LUMO(B) (26%), H-13(B)->LUMO(B) (13%) H-1(A)->L+1(A) (7%), HOMO(A)->LUMO(A) (9%), H-17(B)->LUMO(B) (5%), H-16(B)->L+1(B) (7%)
34	2.6995	459.28	0.1444	H-1(A)->LUMO(A) (10%), HOMO(A)->L+1(A) (12%), H-20(B)->LUMO(B) (26%), H-19(B)->L+1(B) (17%), H-13(B)->L+1(B) (13%), H-17(B)->L+1(B) (5%), H-16(B)->LUMO(B) (9%)
35	2.7222	455.45	0.1253	H-1(A)->L+1(A) (11%), HOMO(A)->LUMO(A) (15%), H-20(B)->L+1(B) (11%), H-19(B)->LUMO(B) (18%), H-17(B)->LUMO(B) (20%), H-16(B)->L+1(B) (12%) H-13(B)->LUMO(B) (3%), H-12(B)->L+1(B) (2%), H-11(B)->LUMO(B) (2%)
36	2.7286	454.39	0.1572	H-1(A)->LUMO(A) (11%), HOMO(A)->L+1(A) (13%), H-20(B)->LUMO(B) (18%), H-19(B)->L+1(B) (14%), H-17(B)->L+1(B) (14%), H-16(B)->LUMO(B) (13%), H-13(B)->L+1(B) (7%)

Table S8. Energy, wavelength, oscillator strength, and composition of the TDDFT excitations of "**po**$^{+2}$" in chlorobenzene.

States	Energy (eV)	Wavelength (nm)	F	Composition
1	0.7675	1615.52	0.0235	H-1(B)->L+1(B) (34%), HOMO(B)->LUMO(B) (66%)
3	0.8326	1489.18	0.6443	H-1(B)->LUMO(B) (34%), HOMO(B)->L+1(B) (65%)
4	0.8481	1461.85	0.8110	H-1(B)->L+1(B) (66%), HOMO(B)->LUMO(B) (34%)
7	1.6936	732.08	0.0339	H-5(B)->L+1(B) (35%), H-4(B)->LUMO(B) (57%), H-3(B)->L+1(B) (4%), H-2(B)->LUMO(B) (2%)
9	1.7155	722.71	0.0364	H-7(B)->L+1(B) (30%), H-6(B)->LUMO(B) (57%) H-7(B)->LUMO(B) (7%), H-6(B)->L+1(B) (3%)
17	2.1179	585.41	0.1074	H-9(B)->L+1(B) (35%), H-8(B)->LUMO(B) (61%)
35	2.7489	451.04	0.3311	H-1(A)->LUMO(A) (19%), HOMO(A)->L+1(A) (23%), H-20(B)->LUMO(B) (27%), H-19(B)->L+1(B) (14%) H-17(B)->L+1(B) (3%), H-16(B)->LUMO(B) (2%), H-15(B)->L+1(B) (4%)
36	2.7514	450.62	0.3468	H-1(A)->L+1(A) (17%), HOMO(A)->LUMO(A) (21%), H-20(B)->L+1(B) (15%), H-19(B)->LUMO(B) (22%) H-15(B)->LUMO(B) (4%), H-14(B)->L+1(B) (9%), H-13(B)->LUMO(B) (3%)
37	2.7546	450.09	0.0623	H-16(B)->L+1(B) (23%), H-15(B)->LUMO(B) (28%), H-14(B)->L+1(B) (24%), H-1(A)->L+1(A) (3%), HOMO(A)->LUMO(A) (4%), H-20(B)->L+1(B) (5%), H-19(B)->LUMO(B) (2%), H-17(B)->LUMO(B) (2%), H-13(B)->LUMO(B) (6%)
38	2.7556	449.94	0.0307	H-16(B)->LUMO(B) (15%), H-15(B)->L+1(B) (42%), H-14(B)->LUMO(B) (24%), H-13(B)->L+1(B) (12%) H-1(A)->LUMO(A) (2%), HOMO(A)->L+1(A) (2%), H-19(B)->L+1(B) (3%)
41	2.8426	436.16	0.2329	H-1(A)->L+1(A) (16%), HOMO(A)->LUMO(A) (21%), H-20(B)->L+1(B) (18%), H-19(B)->LUMO(B) (34%) H-18(B)->L+1(B) (2%)
42	2.8468	435.53	0.3585	H-1(A)->LUMO(A) (17%), HOMO(A)->L+1(A) (22%), H-20(B)->LUMO(B) (29%), H-19(B)->L+1(B) (23%)

Table S9. Energy, wavelength, oscillator strength, and composition of the TDDFT excitations of "**pm^{+2}**" in chlorobenzene.

States	Energy (eV)	Wavelength (nm)	F	Composition
3	0.7433	1667.95	0.5751	H-1(B)->LUMO(B) (10%), H-1(B)->L+1(B) (28%), HOMO(B)->LUMO(B) (26%), HOMO(B)->L+1(B) (36%)
4	0.7756	1598.55	0.8325	H-1(B)->LUMO(B) (35%), H-1(B)->L+1(B) (28%), HOMO(B)->LUMO(B) (10%), HOMO(B)->L+1(B) (27%)
6	1.5044	824.16	0.0257	H-2(B)->LUMO(B) (63%), H-2(B)->L+1(B) (36%)
7	1.5230	814.08	0.0558	H-5(B)->L+1(B) (16%), H-4(B)->LUMO(B) (50%), H-4(B)->L+1(B) (23%) H-5(B)->LUMO(B) (9%)
17	2.1021	589.82	0.0877	H-8(B)->LUMO(B) (63%), H-8(B)->L+1(B) (34%)
18	2.1371	580.15	0.0868	H-9(B)->LUMO(B) (54%), H-9(B)->L+1(B) (43%)
37	2.6984	459.47	0.5702	H-1(A)->L+1(A) (18%), HOMO(A)->LUMO(A) (40%), HOMO(A)->L+1(A) (16%), H-1(A)->LUMO(A) (5%), H-19(B)->LUMO(B) (5%), H-19(B)->L+1(B) (4%)
38	2.7070	458.02	0.6303	H-1(A)->LUMO(A) (31%), H-1(A)->L+1(A) (17%), HOMO(A)->L+1(A) (24%), HOMO(A)->LUMO(A) (6%), H-20(B)->LUMO(B) (6%), H-20(B)->L+1(B) (3%)
39	2.8060	441.85	0.0302	H-19(B)->LUMO(B) (37%), H-19(B)->L+1(B) (33%) HOMO(A)->LUMO(A) (3%), HOMO(A)->L+1(A) (2%), H-20(B)->L+1(B) (2%), H-18(B)->LUMO(B) (5%), H-17(B)->L+1(B) (5%)
42	2.8241	439.02	0.0309	H-20(B)->LUMO(B) (41%), H-20(B)->L+1(B) (23%) H-1(A)->LUMO(A) (2%), HOMO(A)->L+1(A) (2%), H-19(B)->L+1(B) (3%), H-18(B)->LUMO(B) (5%), H-18(B)->L+1(B) (5%), H-17(B)->LUMO(B) (3%), H-17(B)->L+1(B) (3%)
